# Increased expression of the immunoproteasome subunits PSMB8 and PSMB9 by cancer cells correlate with better outcomes for triple-negative breast cancers

**DOI:** 10.1038/s41598-023-28940-2

**Published:** 2023-02-06

**Authors:** Karen Geoffroy, Bruna Araripe Saraiva, Melissa Viens, Delphine Béland, Marie-Claude Bourgeois-Daigneault

**Affiliations:** 1 Cancer and Immunopathology Axes, CHUM Research Centre, Montreal, Canada; 2 Department of Microbiology, Infectious Diseases and Immunology, Faculty of Medicine, University of Montreal, Montreal, Canada; 3 Institut du Cancer de Montréal, Montreal, Canada

**Keywords:** Breast cancer, Cancer, Cancer microenvironment

## Abstract

Proteasome dependency is a feature of many cancers that can be targeted by proteasome inhibitors. For some cancer types, notably breast cancer and triple-negative breast cancer (TNBC), high mRNA expression of a modified form of the proteasome, called the immunoproteasome (ImP), correlates with better outcomes and higher expression of one ImP subunit was associated with slower tumor growth in a small patient cohort. While these findings are in line with an anti-tumoral role of the ImP in breast cancer, studies investigating ImP expression at the protein level in large patient cohorts are lacking. Furthermore, while ImPs can be found in both immune and non-immune cells, the cellular source is often ignored in correlative studies. In order to determine the impact of ImP expression on breast cancer outcomes, we assessed the protein expression and cellular source of the ImP subunits PSMB8 and PSMB9 in a cohort of 2070 patients. Our data show a clear correlation between high ImP expression and better outcomes, most notably for TNBC patients and when tumor cells rather than stromal or immune cells express PSMB8 or PSMB9. Our results therefore suggest that ImP expression by tumor cells could be used as prognostic markers of TNBC outcomes.

## Introduction

Breast cancer is a heterogeneous disease that accounts for 30% of newly diagnosed cases and 15% of cancer-related deaths in American women^1^. It can be classified into intrinsic molecular subtypes based on the expression of different genes, including the receptors for estrogen (ER), progesterone (PR) and human epidermal growth factor (HER-2). As such, luminal breast cancers are ER+, PR+, HER-2- and express genes of luminal breast cells, whereas HER-2-positive breast cancers overexpress HER-2. Finally, basal-like breast cancers express genes found in basal breast cells and are usually negative for ER, PR and HER-2^2^. Basal-like breast cancers have the worst outcomes and affect younger women^3^.

Basal-like breast cancers are heterogeneous, and while most of them (77%) are triple-negative breast cancers (TNBC) that do not express ER, PR or HER-2, a fraction express at least one of the receptors and are therefore not TNBCs^4^. Reciprocally, not all TNBCs express basal breast cell genes and thus only 71% are also basal-like^5^. Compared to other breast cancers, TNBCs affect younger women, are particularly aggressive and lack effective treatment options. Although some TNBC patients treated at early stages respond well to cancer immunotherapies, advanced forms of the disease are refractory; therefore, the effective treatment of TNBCs remains a clinically unmet need^6^. While many treatment modalities are currently being explored for these patients, curative options and prognostic markers predictive of outcomes and patient responses to treatment are urgently needed.

One particularity of TNBC is its proteasome dependency. Indeed, proteasome genes were found to be particularly important for TNBC cell survival compared to other cancer types and proteasome inhibitors were found to be effective against the disease in mice^7^. Proteasomes are protein complexes found in all living organisms and for which 3 subunits have proteolytic activity. Specialized immune cells, as well as other cells exposed to inflammatory conditions, have the capacity to induce and assemble a modified form of the proteasome called the immunoproteasome (ImP), for which the 3 proteolytic subunits are substituted for alternative ones with different cleavage abilities^8^. Thus, the proteolytic subunits PSMB (Proteasome subunit beta type) 1, 2 and 5 of the proteasome are replaced by PSMB9, PSMB10 and PSMB8, respectively, in the ImP^8^. PSMB8 and 9 both have chymotrypsin-like activity, while PSMB10 performs proteolysis in a trypsin-like manner^8^. The chymotrypsin-like cleavage is particularly important in the context of antigen presentation as it allows for the generation of peptides with the proper anchor residue to fit and bind into MHC-I (major histocompatibility complex class I) pockets^8^. Interestingly, a recent study by the group of Craik has shown that the repertoire of peptides generated by the constitutive proteasome versus the ImP were only partially overlapping. It is noteworthy however that the generation of peptides with MHC-I binding capacity was unaffected^9^. Differences in peptide repertoires are believed to affect both the induction of an immune response, as well as the immune recognition of target cells. The ImP plays important roles in both immune and non-immune cells and its function contributes to autoimmune diseases, as well as processes such as cytokine production and the regulation of NF-κB activation^10^.

Cancer cells have increased proliferation rates, which require faster protein synthesis and therefore elevated proteasome activity^11^. Additionally, high proteasome activity contributes to cell survival and tumorigenesis by preventing apoptosis and clearing damaged proteins^12^. Interestingly, proteasome addiction was found to be a characteristic of most aggressive and drug-resistant cancers, including TNBCs^13^. Blocking proteasome degradation using inhibitors (Bortezomib, Carfilzomib and Ixazomib) has been pursued therapeutically, but was mostly effective against blood cancers and results were disappointing in the solid cancer setting^14–17^.

While both proteasome and ImP genes are overexpressed in most cancers as determined from the Cancer Genome Atlas, increased ImP expression has opposite effects in different cancer types^14,18^. For example, the ImP inhibitor ONX 0914 was found to be effective against colon cancer^19^, myeloma^20^ and acute lymphoblastic leukemia^21^ in mice, therefore indicating a pro-tumoral role of the ImP in these malignancies. In line with this, increased PSMB8 expression correlates with the aggressiveness of gliomas^22^, the carcinogenesis of renal cell carcinoma^23^ and is associated with poor outcomes in gastric cancers^24^. For breast cancer specifically, the opposite was found: high ImP mRNA levels are correlated with longer survival and seem to be influenced by tumor-infiltrating lymphocytes (TILs)^18,25^. Also, for a small cohort of breast cancer patients, estrogen receptor alpha-negative tumors (which include TNBCs) for which PSMB9 expression was elevated had slower tumor growth^26^. Increased ImP expression was also associated with patient benefits for other cancers. For example, high PSMB8 and PSMB9 expression correlates with better outcomes and response to immune checkpoint inhibitors for melanoma patients^27^ and PSMB8 overexpression in non-small cell lung cancer is associated with fewer cases of relapse and metastasis^28^, as well as better 5 years survival rates^29^. Furthermore, in support of a role of the ImP in cancer immunosurveillance, mice lacking PSMB9 expression spontaneously develop uterine leiomyosarcoma^30^.

Most studies investigating ImP expression focus either on gene overexpression, a single ImP subunit, or total expression within heterogeneous tumor samples. Here, we sought to determine the impact of PSMB8 and PSMB9 expression at the protein level on outcomes in breast cancer and TNBC patients, but also in the context of the cell type expressing the ImP proteins. Our results show significant benefits, notably in terms of relapse-free survival (RFS), as well as metastasis to bones and liver, with increased expression of both PSMB8 and PSMB9 by tumor cells, particularly in the context of basal-like breast cancer and TNBC.

## Results

### Higher transcript expression of the ImP subunits PSMB8 and PSMB9 is associated with better prognosis in basal-like and TNBC patients

**Figure 1:**
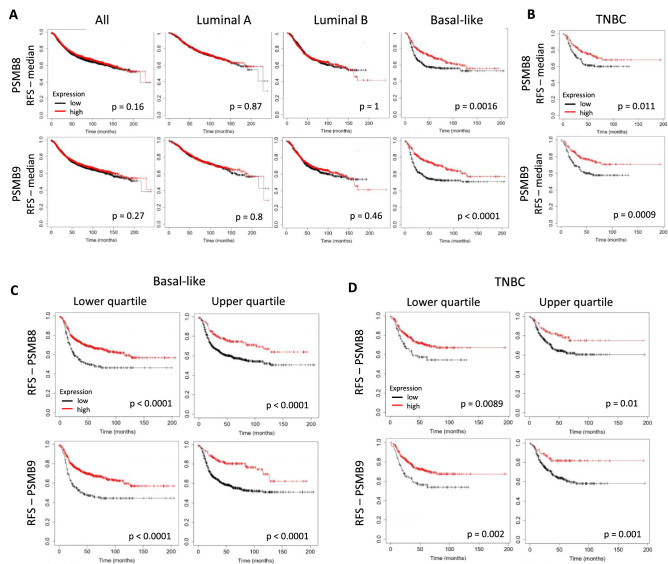
High PSMB8 and PSMB9 mRNA expression is associated with better prognosis in basal-like breast cancer and TNBC. RFS according to PSMB8 and PSMB9 expression of (**A**) all breast cancer patients, as well as for the luminal A, luminal B, basal-like breast cancer or (**B**) TNBC subtypes specifically. The groups were divided according to their median mRNA expression. The same analysis was performed according to upper and lower quartiles of expression for (**C**) basal-like breast cancer, as well as (**D**) TNBC patients. The red and black lines correspond to high and low expression, respectively. Statistical analyses by Logrank test: differences are considered statistically significant when *p* < 0.05.

To determine if ImP expression is linked to outcomes of breast cancers, we first analyzed publicly available microarray datasets from 4929 breast cancers of all subtypes (Kaplan–Meier plotter)^31^. We focused our analysis on the PSMB8 and PSMB9 catalytic subunits because they are the ones with chymotrypsin-like activity and have been the most often associated with disease progression and outcomes. Our analysis revealed no advantage to either PSMB8 or PSMB9 overexpression when considering all breast cancer subtypes together, as well as for the luminal A or luminal B subtypes specifically (Fig. 1A). Strikingly, we found the RFS to be extended for both genes when focusing on the basal-like breast cancer subtype alone (Fig. 1A). Basal-like breast cancers include TNBCs, a particularly aggressive and deadly form of the disease. When analyzing TNBC samples from the same cohort, we once again found a significant advantage to high PSMB8 and PSMB9 expression (Fig. 1B). Our findings were similar when assessing the overall survival (OS) with high PSMB8 and PSMB9 mRNA expression being particularly important for basal-like breast cancers and important but not statistically significant for TNBC (Fig. S1). To further investigate the link between RFS and PSMB8/PSMB9 mRNA expression, we segregated basal-like and TNBC samples into quartiles of expression (Fig. 1C,D). The higher and lower quartiles represent the top and bottom 25% of samples with the highest and lowest mRNA expression values, respectively. Quartiles include 101/404 samples for TNBC and 212/848 samples for basal-like breast cancers. For both indications, we found the expression levels to correlate with relapse, with higher expression corresponding to better outcomes.

Given the reported link between ImP expression and immune cell infiltration in breast cancer^25^, we repeated our RFS analysis using the same patient cohort, but this time segregating samples according to CD45 expression. We once again observed the most advantage to high CD45 expression for basal-like and TNBC patients, but this time also when considering all breast cancer subtypes, as well as luminal B samples (Fig. S2A). Although the same trend was observed when assessing the OS, the difference was only statistically significant for all breast cancers, as well as for basal-like breast cancers (Fig. S2B).

### Higher frequency of PSMB8- or PSMB9-expressing cells is associated with better outcomes in TNBC patients

While the RNA expression of PSMB8 and PSMB9 is informative, the protein expression is more relevant when considering post-transcriptional regulation, as well as ImP function. We therefore performed our analysis at the protein level. To do so, we took advantage of another breast cancer patient cohort. The Quebec Breast Cancer Foundation (QBCF) cohort includes a paraffin-embedded tissue microarray generated from 2070 breast cancer samples of all types, as well as clinical data (details in Supp. Table 1). In order to measure the protein expression of the ImP subunits, we designed immunofluorescence (IF) panels that allow for the co-staining of PSMB8 or PSMB9 together with cytokeratins 8 and 18 (CK8-18) that mark epithelial (tumor) cells, CD45 to identify immune cells and DAPI to detect nuclei from all cells. Given that many immune cells constitutively express the ImP, we first optimized our panels on human lymph node samples. As expected, most cells were positive for CD45 and both PSMB8 and PSMB9, and CK8-18 did not label any cells (Fig. S3A,B). To confirm the CK8-18 stain, we used a human breast cancer sample. As expected, epithelial cells stained positive for CK8-18, therefore confirming the validity of our panel (Fig. S3C). The specificity of the stains was validated by a pathologist. Next, to confirm that PSMB8 and PSMB9 antibodies both recognized their target proteins, we took advantage of knockout cell lines that we have in-house. The cells were treated with interferon (IFN)-gamma to induce ImP expression and the samples were probed with either PSMB8 or PSMB9 antibodies. Our data show the loss of signal in the knockout cell lines, therefore confirming the specificity of our antibodies (Fig. S3D,E).

**Figure 2:**
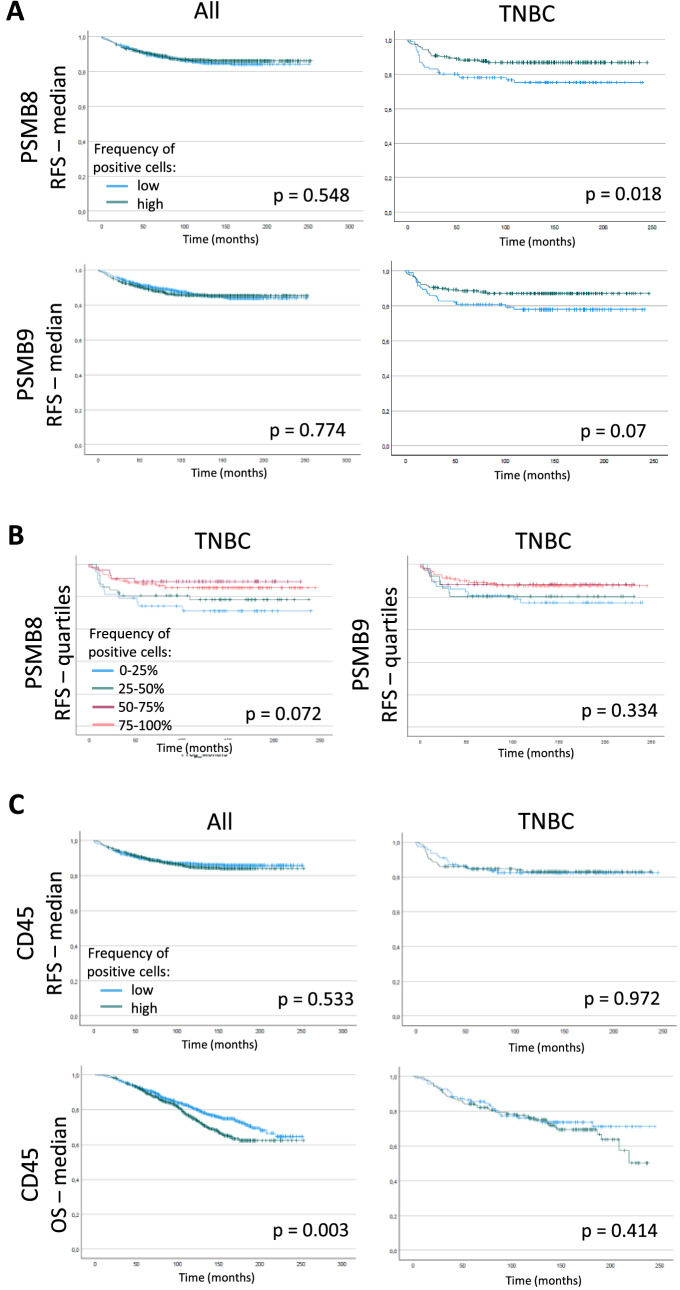
Overall high PSMB8 and PSMB9 protein expression is associated with a trend to better outcomes in TNBC patients specifically. RFS according to the amount of PSMB8- and PSMB9-expressing cells of all breast cancer subtypes together as well as TNBC patients divided according to (**A**) their median or (**B**) by quartiles of expression. (**C**) The same analysis was performed for the RFS, as well as the OS of the patients according to the median CD45 protein expression. For median expression, the green and blue lines correspond to high and low frequency of positive cells, respectively; for quartiles expression, blue, green, purple and red lines correspond to the lowest to highest frequency of positive cells, respectively. Statistical analyses by Logrank test: differences are considered statistically significant when *p* < 0.05.

We then applied our IF panels to the breast cancer tissue microarray and first quantified the number of PSMB8- or PSMB9-positive cells in each sample. We found no difference in RFS when analyzing all breast cancer subtypes together (Fig. 2A, left panels). We then focused on the TNBC population. Given the low number of TNBC samples, and to ensure a proper representation of the range of cells that can be found within breast cancers, we segregated TNBC into quartiles based on the values calculated for all breast cancer types (details in Supp. Table 2). When focusing on TNBC patients, high frequency of PSMB8- or PSMB9-expressing cells both correlated with better outcomes, although the results were only statistically significant for PSMB8 (Fig. 2A, right panels). When segregating patients into quartiles of frequency of ImP-expressing cells, we found that, although once again not statistically significant, outcomes were better when more cells expressed either PSMB8 or PSMB9 (Fig. 2B). Importantly, we also found higher numbers of PSMB8- and PSMB9-expressing cells to correlate with better RFS when dividing samples according to medians and quartiles of values that are specific to TNBC samples (Fig. S4). We then analyzed the impact of ImP expression on OS, as well as bone, brain, liver, lungs, skin and lymph node metastasis, and found that a high frequency of PSMB8-expressing cells correlated with fewer patients with bone and liver metastases, and a trend to fewer patients with brain metastases (Fig. S5A, right panels), while PSMB9 expression had no significant correlations for all breast cancer patients together (Fig. S5B, left panel). Also, although not statistically significant, fewer TNBC patients with more cells expressing PSMB9 had bone, brain and liver metastases (Fig. S5B, right panel).

We then assessed if CD45-positive (immune) cells were indicative of outcome. As opposed to the positive correlation we measured using the KMplotter database^31^ at the mRNA level, we found a negative correlation between the number of immune cells and OS when analyzing all breast cancer patients together (Fig. 2C). We also found no link with the RFS or OS for TNBC samples (Fig. 2C). Interestingly, while the quantities of PSMB8- or PSMB9-positive cells increased significantly with the grade of the disease, the number of immune cells remained the same for grade 1 and 2 breast cancers and decreased for grade 3 (Fig. S6). Furthermore, when analyzing whether CD45 and PSMB8 or PSMB9 expression were linked, we found an inverse correlation (Fig. S7), therefore suggesting that increased ImP expression is not positively linked to immune cell infiltration in our breast cancer patient cohort.

### High frequency of PSMB8- or PSMB9-expressing tumor cells is associated with better prognosis in TNBC patients

**Figure 3:**
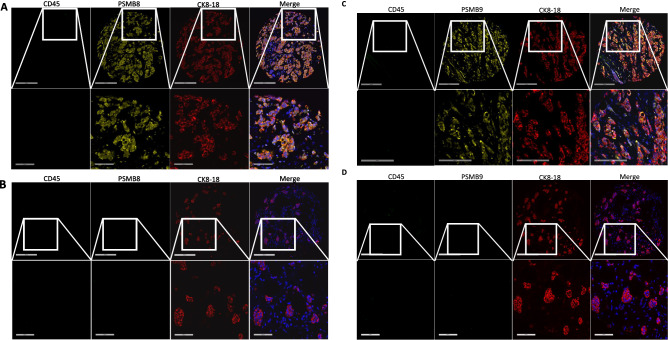
Breast cancer samples have heterogeneous patterns of PSMB8 and PSMB9 expression. IF images of breast cancer samples stained with combinations of (**A**) and (**B**) PSMB8, CD45, CK8-18 and DAPI or (**C**) and (**D**) PSMB9, CD45, CK8-18 and DAPI. Panels (**A)** and (**C)** are samples representative of high ImP expression and panels (**B**) and (**D**) of low or no ImP expression. Upper panels show the whole sample cores and lower panels are magnifications of the regions delineated by white squares. DAPI (blue), PSMB8 or PSMB9 (yellow), CD45 (green), CK8-18 (red). Whole core scale bar = 300 µm, close-up scale bar = 200 µm (**A**) or 100 µm (**B**–**D**).

**Figure 4:**
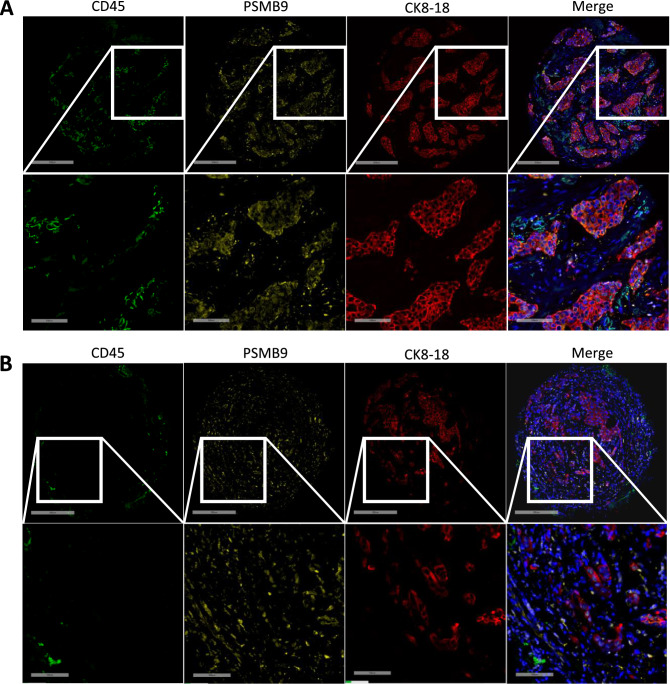
Breast cancer samples have heterogeneous degrees of CD45-positive cells infiltration. IF images of breast cancer samples stained with PSMB9 (yellow), CD45 (green), CK8-18 (red) and DAPI (blue). The images show representative samples containing (**A**) high or (**B**) low CD45-positive cells infiltration. Upper panels show the whole sample cores and lower panels are magnifications of the regions delineated by white squares. Whole core scale bar = 300 µm, close-up scale bar = 100 µm.

**Figure 5:**
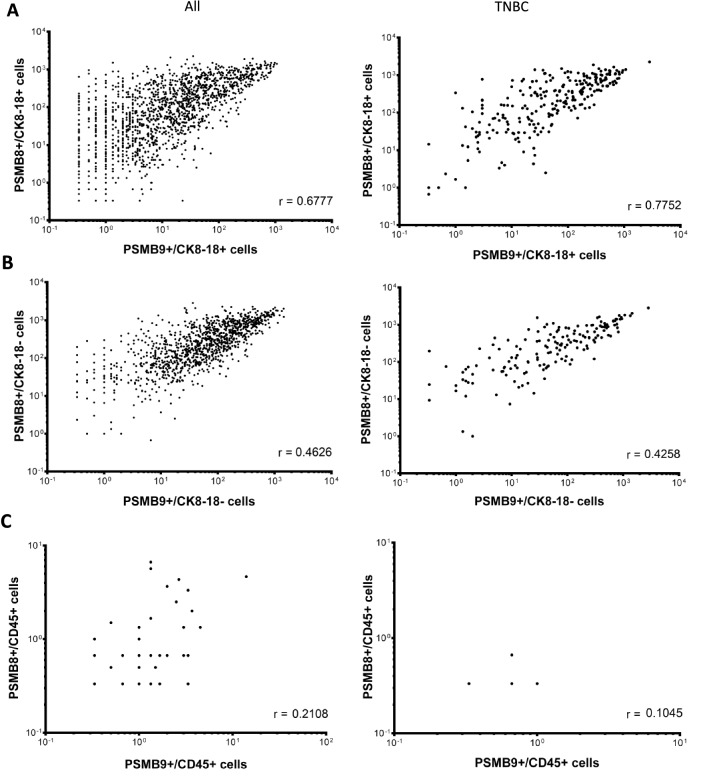
Numbers of PSMB8- and PSMB9-expressing cells correlate for all breast cancers and TNBC samples. Linear correlation regression analysis of protein expression for PSMB8 and PSMB9 in (**A**) tumor cells (CK8-18), (**B**) stromal cells (CK8-18-) or (**C**) immune cells (CD45+) in all breast cancer (left panel), as well as TNBC (right panel) samples. Spearman's rank correlation coefficient indicates a correlation when r ≥ 0.2.

**Figure 6:**
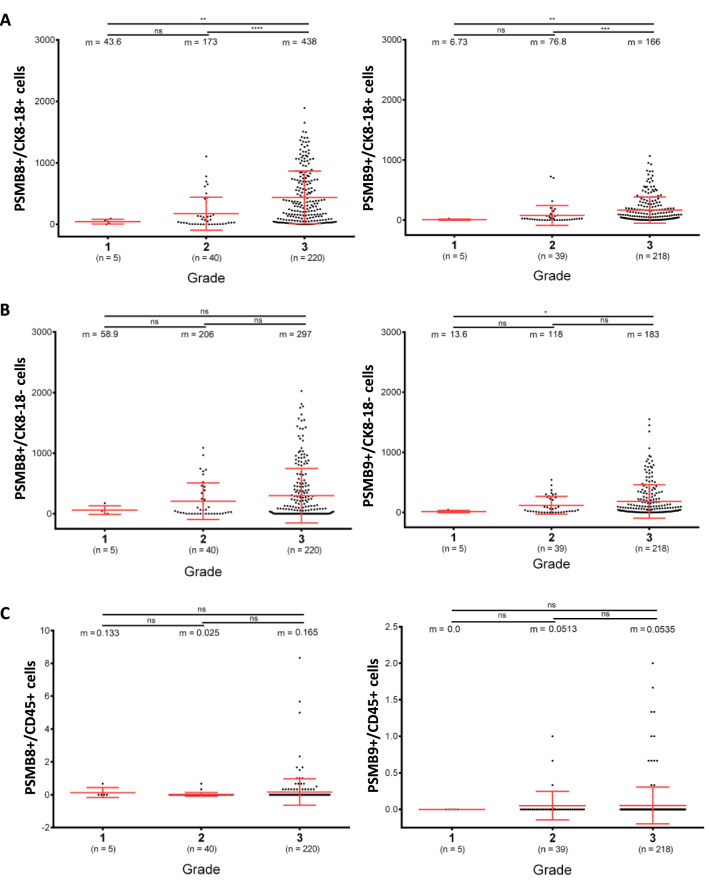
Protein expression of PSMB8 and PSMB9 by tumor cells correlate with breast cancer grades. Quantification of the number of (**A**) tumor cells (CK8-18+), (**B**) stromal cells (CK8-18−) or (**C**) immune cells (CD45+) positive for PSMB8 (left panel) or PSMB9 (right panel) and segregated according to the grade of TNBC cancers. The number of samples from each grade included in the analysis is indicated in brackets below the corresponding group. Mann-Whitney test, ns: *p* > 0.05, *p ≤ 0.05, **p ≤ 0.01, ***p ≤ 0.001, ****p ≤ 0.0001.

**Figure 7:**
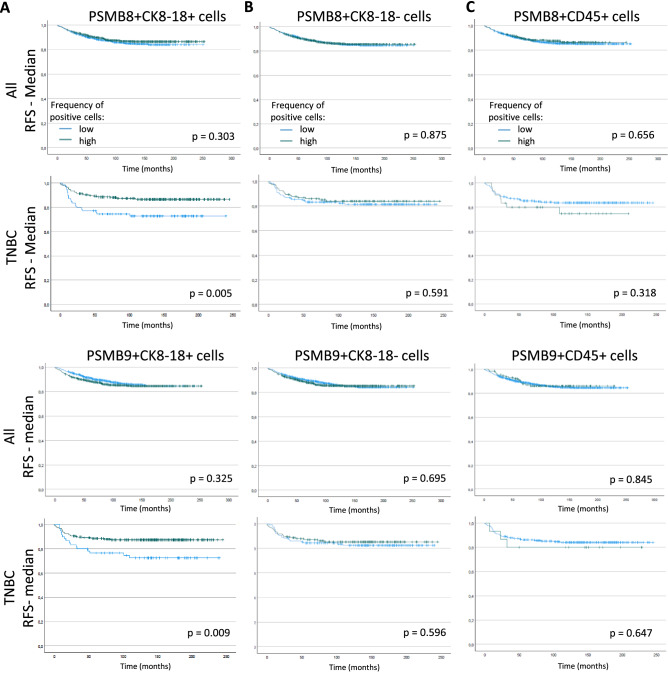
Protein expression of PSMB8 and PSMB9 in tumor cells is associated with better prognosis in TNBC samples. RFS according to PSMB8 (upper panel) and PSMB9 (lower panel) expression by (**A**) tumor cells (CK8-18+), (**B**) stromal cells (CK8-18−) or (**C**) immune cells (CD45+) of all breast cancer subtypes together and TNBC samples. For (**A**) and (**B**), samples were divided according to their median number of cells and for (**C**), according to the presence or absence of CD45-expressing cells. The green and blue lines correspond to high and low frequency of positive cells, respectively. Statistical analyses by Logrank test: differences are considered statistically significant when *p* < 0.05.

**Figure 8:**
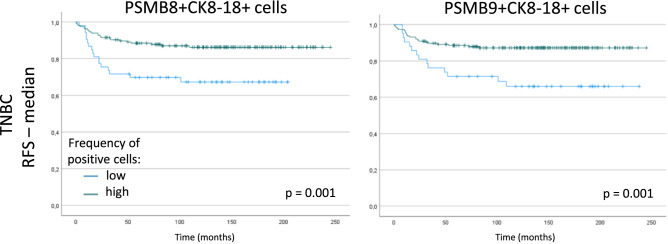
Protein expression of PSMB8 and PSMB9 in tumor cells is associated with better RFS in high-grade TNBC patients. RFS according to PSMB8 and PSMB9 expression by tumor cells (CK8-18+) of grade 3 TNBC samples. Samples were divided according to their median number of cells. The green and blue lines correspond to high and low frequency of positive cells, respectively. Statistical analyses by Logrank test: differences are considered statistically significant when *p* < 0.05.

In addition to the number of cells expressing the different proteins, our antibody panels also allowed us to identify which cells express PSMB8 and PSMB9. We observed heterogeneous patterns of expression across our samples. As such, some tumors contained high amounts of PSMB8+ CK8-18+ (tumor) cells, while some others were completely negative (Fig. 3A,B) and the same phenotypes were observed for PSMB9 (Fig. 3C,D). Next, we wanted to determine if the samples that contained more PSMB8 + cells also contained more PSMB9 + cells and found a strong correlative association for all breast cancer subtypes together, as well as for TNBC specifically (Fig. S8). When analyzing the frequency of immune cells, we once again found samples that contained high numbers of these cells (Fig. 4A) and others that were poorly infiltrated with immune cells (Fig. 4B). We then analyzed the relationships between PSMB8- versus PSMB9-expressing tumor cells (CK8-18+, CD45-), stromal cells (CK8-18-, CD45-) and immune cells (CK8-18-, CD45+). We found strong positive correlations for all breast cancers together and TNBC patients specifically for tumor cells and stromal cells, as well as for immune cells of all breast cancer subtypes, but not for TNBC patients (Fig. 5A–C). We next examined if the amounts of each cell type expressing PSMB8 or PSMB9 varied according to the grade of the disease. Our results show that PSMB8- and PSMB9-expressing tumor cells increased with grades (Fig. 6A), while PSMB9-expressing stromal cells increased only between the lowest and the highest grades of our cohort (Fig. 6B, right panel) and the amount of PSMB8-positive stromal cells, as well as PSMB8- and PSMB9-expressing immune cells did not vary according to the grade of the disease (Fig. 6B, left panel and C). We then analyzed the outcomes of breast cancer and TNBC patients based on the types of cells expressing PSMB8 and PSMB9. Once again, the TNBC groups were divided according to ranges of cell numbers detected for all breast cancers. We found an advantage for patients with high numbers of positive cells only when the cells expressing the ImP subunits were tumor cells and specifically for TNBC patients (Fig. 7A). This was also observed when dividing the groups according to TNBC-specific median and quartile values (Fig. S9). Furthermore, this was specific to tumor cells since the amount of immune cells or stromal cells expressing PSMB8 or PSMB9 were not associated with differences in RFS (Fig. 7B,C). When looking at the OS and metastases, significant associations were found for PSMB8-expressing tumor cells and OS, as well as PSMB9-expressing tumor cells and brain metastasis (Fig. S10A). No significant differences were found for the number of PSMB8- or PSMB9-expressing stromal or immune cells (Fig. S10B, C). Given that the amounts of PSMB8- and PSMB9-expressing cells increase with grades (Figs. 6 and S5), but correlate with prolonged RFS in TNBC (Figs. 2 and 7), we next wanted to determine if amongst higher-grade cancers, the benefits of PSMB8/9 expression are even greater. As expected, the benefits of higher PSMB8- and PSMB9-positive tumor cells content were augmented when considering high-grade cancers only (Fig. 8). Indeed, when considering all TNBC grades, the RFS at 240 months corresponded to 73.4% (PSMB8) and 73.3% (PSMB9) versus 87.2% (PSMB8) and 87.7% (PSMB9) for the low and high expressers, respectively (Fig. 7A). When considering only grade 3 TNBCs, these numbers changed to 67.9% (PSMB8) and 66.7% (PSMB9) versus 86.8% (PSMB8) and 87.5% (PSMB9) for the low and high expressers, respectively (Fig. 8). Therefore, amongst high grade TNBCs, 20% more patients with high content of ImP subunit-expressing tumor cells survived longer without relapse compared with patients with fewer of these cells.

## Discussion

Our study demonstrates an advantage to high expression of the ImP subunits PSMB8 and PSMB9 and high frequencies of ImP-expressing cells for TNBC patient outcomes. Our results are in line with previous reports showing the slower growth of estrogen receptor-negative breast cancers with high PSMB9 expression^26^ and another one demonstrating that increased ImP gene expression is associated with longer survival for breast cancer patients^25^. While some associations show trends that are consistent but not statistically significant when looking at TNBC patients only, we believe that this is because of the low number of TNBC patients in our cohort (282). The finding that ImP expression benefits mostly basal-like breast cancers and TNBCs rather than all breast cancer subtypes is interesting. Given the important role of the ImP for antigen presentation, this difference could be the result of tumor control by immune cells. In line with this possibility, basal-like breast cancers and TNBCs were found to have increased mutational burdens compared to other breast cancer types^32,33^. Thus, increased ImP expression in the context of more abundant tumor-specific mutations could allow for enhanced presentation of tumor antigens at the cell surface and immune-mediated control of the disease.

Interestingly, while our results show an inverse correlation between the number of CD45-positive and PSMB8- or PSMB9-expressing cells, a previous study found PSMB8 expression by both breast cancer and TNBC to correlate with the amount of tumor-infiltrating lymphocytes (TILs)^18^. While these findings appear contradictory, CD45 is a membrane-bound glycoprotein found on all hematopoietic cells and is therefore used to distinguish immune from non-immune cells^34^. Thus, our IF panels label CD45-positive cells include TILs but also stain other immune cells such as dendritic cells, macrophages and myeloid-derived suppressor cells, which can all be found in breast tumors^35^. The authors of the previous study identified an IFN signature as also correlating with PSMB8 expression and suggest that TILs, which only include lymphocytes, are responsible for this signature. In our analysis, immune-suppressive immune cells such as M2 macrophages and myeloid-derived suppressor cells, which are also CD45+, were also taken into account and would not contribute to an IFN signature as was shown by the authors of the other study. In order to determine the specific importance of TILs (rather than all immune cells as we did) for PSMB8 expression in our patient cohort, we would have to repeat our analysis with a CD3-specific antibody. Our conclusion that immune cell infiltration does not correlate with PSMB8- and PSMB9-expressing cells therefore remains valid and is not in conflict with the finding that they correlate with TILs.

Interestingly, our results show a positive correlation between CD45 mRNA expression and outcomes, but we also found a negative correlation between the frequency of CD45-positive cells and OS in a different patient cohort when analyzing all breast cancers together. This difference could be explained by the fact that different immune cell populations express various levels of CD45. For instance, amongst lymphocytes, NK T cells are the ones that express the highest levels of CD45, while B cells express the lowest^36^. Therefore, the mRNA analysis of CD45 expression could be misleading depending on the immune cell types that are found in the tumors whereas the frequency of CD45-expressing cells reflects better the immune cell content in terms of amounts.

In the same study, Lee et al.^18^ found PSMB8 expression to not be associated with grades whereas our results show that the numbers of both PSMB8- and PSMB9-expressing cells increase with grade. It should be noted that we analyzed the amount of PSMB8- and PSMB9-expressing cells, which is different from the other study that considered the overall level of protein expression. Once again, these results are not necessarily contradictory. For instance, lower expression of the protein in more cells versus fewer cells expressing very high levels of PSMB8 could explain both results. Although we did not analyze the overall level of protein expression in our cohort, it is possible that the number of cells expressing PSMB8 or PSMB9, but not the overall level of expression throughout the sample, increases with tumor grade.

Importantly, we found that it was ImP expression specifically within tumor cells that conferred the most benefits. While most cells can induce PSMB8 and PSMB9 in inflammatory conditions such as with IFN or tumor necrosis factor alpha (TNFα) stimulation or in the case of oxidative stress^8^, non-immune cells do not usually express ImPs at the steady state. Tumor cells can induce the ImP for different reasons, for instance as a consequence of immune cell infiltration or treatments that trigger inflammation. Interestingly, our results show the number of PSMB8- and PSMB9-positive cells increase with increasing grade, and while higher grades usually have worst outcomes, we still observed considerable benefits to high numbers of ImP-expressing tumor cells with almost 15% of patients surviving longer. Excitingly, when focusing the analysis on grade 3 cancers only, we found an even greater advantage to higher content of ImP-positive tumor cells with 20% of TNBC patients having an extended RFS. This finding highlights the potential of using PSMB8- and PSMB9-positive tumor cells as prognostic factors.

Several treatment modalities that cause inflammation and therefore induce or might induce ImP expression have the potential to confer benefits to cancers like basal-like breast cancer and TNBC for which ImP expression is indicative of good outcomes. Furthermore, given that it has been demonstrated that high ImP expression could improve responsiveness to cancer immunotherapies such as immune checkpoint blockade for melanoma^27^, inducing ImP expression in ImP-low TNBC has the potential to add treatment options for these patients that have a poor prognosis. As such, cisplatin, paclitaxel and 5-fluorouracil^37^, as well as doxorubicin^38^, are all drugs already approved for the treatment of different cancers, including breast cancer and TNBC, and that have been shown to induce inflammation. While treatment-induced inflammation is usually associated with drug-associated toxicities, it might be nonetheless beneficial to induce ImP expression, and may point to a previously unknown mechanism by which these drugs are effective against cancer. A novel immunotherapeutic cancer treatment option that triggers inflammation, notably by the production of IFNs, is oncolytic virotherapy^39^. Our group has shown in many studies that several of these cancer-killing viruses were promising treatment options against TNBC^40–43^ and could induce ImP expression^42^. Clinically, our findings could help guide the choice of treatment of TNBC patients for ones that induces ImP expression, as well as combinations of these treatments with immune checkpoint blockade to which TNBCs usually respond poorly.

We found that PSMB8 or PSMB9 expression by tumor cells could be used as biomarkers in the clinic to predict TNBC prognosis, but whether both markers together would confer additional information or would be even more sensitive to predict outcomes remains to be determined. Given that we used two separate antibody panels to detect PSMB8 and PSBM9, we cannot answer this question. However, we found a strong correlation between the numbers of PSMB8 + and PSMB9 + cells, as well as between PSMB8 + and PSMB9 + tumor cells, which would suggest that using only one or the other could potentially be sufficient. While this study would suggest the prioritization of PSMB8 further studies are needed to fully understand the potential of PSMB9. Another limitation of our study is the limited number of TNBC samples in our cohort, which may contributed to some differences not being statistically significant. Future work will aim at using a patient cohort that includes more TNBC samples or extending the current cohort as well as staining both PSMB8 and PSMB9 in the same panel to overcome these limitations.

## Conclusion

In this study, we found that the presence of ImP-expressing tumor cells is predictive of better outcomes for TNBC patients. Our results are in line with mRNA expression from publicly available datasets, as well as with previous studies by other groups. Here, not only did we validate these findings at the protein level in a large patient cohort, but we also for the first time characterized the cellular source of PSMB8 and PSMB9 expression, as well as the number of cells expressing the proteins. Our data show the prognostic value of measuring PSMB8- and PSMB9-expressing tumor cells and open the possibility of therapeutically inducing ImP expression to improve outcomes of TNBC patients.

## Material and methods

### Kaplan–Meier analysis of mRNA expression from Kaplan–Meier plotter

Breast cancer survival was analyzed using the Kaplan-Meyer plotter database. mRNA (microarray) of PSMB9 (Affymetrix ID: 204279_at), PSMB8 (209040_s_at) and CD45 (212588_at) were analyzed using this cohort. We performed our analyses on subtypes that were either available as separate categories (basal-like breast cancer, luminal A, luminal B), or that we manually defined according to the expression of different receptors. Kaplan–Meier plotter classifies all ER- and HER-2-negative samples as basal-like breast cancers. For TNBC, we selected samples that were ER- and PR-negative by immunohistochemistry, as well as HER-2 negative by array. Reference for Kaplan–Meier plotter: Lánczky and Győrffy^31^. Available from: http://www.kmplot.com/analysis.

### Breast cancer tissue microarray

The Quebec breast cancer foundation (QBCF) cohort tissue microarray was constructed beginning in 2019. It includes samples from 2070 breast cancer patients which specimens were collected from 2005 to 2014. Triplicate punches were collected from each sample and randomly distributed on the same slide. The complete tissue microarray includes a total of 21 slides and was reviewed by a certified pathologist. Punches are 600 µm wide and 200 µm apart. Patients were followed for at least 2 years if no progression and up to 253 months. All information about the samples can be found in Supplementary Table 1. Briefly, median age of patients: 58, diverse morphologies. According to the Nottingham Histologic Score, 352, 1, 939 and 776 patients were grade 1, 2 and 3, respectively. Samples were also assessed for their expression of HER2, ER and PR. The cohort includes 282 TNBC (ER-, PR- and HER-2-), 113 HER2 + ER-, 178 HER2 + ER + and 1338 ER + HER2-.

This study was approved by the CHUM Research Centre’s ethics committee and experiments were carried out in accordance with institutional guidelines. Informed patient consent was obtained as part of the biobanking process.

### Immunofluorescence

Multi-color IF stains were used to quantify PSMB8, PSMB9 as well as CD45 expression and to delineate the epithelium (containing tumor cells) as well as nucleus. Two separate panels were used: PSMB9, CD45, CK8-18 and DAPI as well as PSMB8, CD45, CK8-18 and DAPI. Antigen retrieval was performed using the Cell Conditioning #1 solution for 60 min with the Benchmark XT autostainer (Ventana Medical System Roche, Oro Valley, AZ). Slides were then incubated for 1 h with a mix of: anti-PSMB9 or -PSMB8, and anti-CD45 antibodies (rabbit anti-human PSBM9 (ab3328 diluted 1:1000 in phosphate-buffered saline (PBS)), rabbit anti-human PSMB8 (ab3328 diluted 1:250 in PBS) and mouse anti-human CD45 (ab8216 diluted 1:500 in PBS) all from Abcam (Cambridge, UK) at room temperature (RT). Slides were then washed in PBS and incubated with the protein-block from Agilent (X0909, Agilent, Santa Clara, CA) for 20 min, incubated for 1 h in a humid chamber at RT with secondary antibodies diluted in a solution of 1% bovine serum albumin (BSA)/PBS (goat anti-rabbit Alexa Fluor 546 (A11010) diluted 1:250 and goat anti-mouse Alexa Fluor 488 (A11001) diluted 1:250, both from Thermofisher Scientific, Waltham, MA) and washed 3 times 10 min in PBS. Slides were then blocked overnight in a humid chamber at 4 °C with a mouse-on-mouse blocking reagent (1 drop in 10 mL of PBS (MKB-2213), Vector Laboratories, Burlingame, CA). The next day, slides were incubated for 1 h with monoclonal mouse anti-human CK8-18 antibodies (MA5-14428 diluted 1:100, Thermofisher and sc-6259 diluted 1:100, Santa Cruz Biotechnology, Dallas, TX, respectively) in a humid chamber at RT, washed in PBS and incubated with goat anti-mouse Cyanine 5 (A31571 diluted 1:250, ThermoFisher Scientific) for 33 min in a humid chamber at RT. A DAPI counterstain solution diluted 1:5000 in PBS (d-3571, Thermofisher scientific) was then added for 7 min. Slides were washed 3 times 5 min in PBS and incubated for 15 min with a sudan black B solution 0.1% (199664 diluted in ethanol 70%, Sigma Aldrich, Saint-Louis, MO). After a final wash in PBS, slides were mounted using fluoromount aqueous mounting medium (f4680, Sigma Aldrich), stored at 4 °C overnight and scanned using an Olympus optical microscope (BX61VSF, Olympus, Tokyo, Japan). Negative control slides (no primary antibodies) were stained in parallel for each experiment. A human tonsil, as well as a ER+, PR+, HER2- breast cancer sample were used for the validation of our IF panels (Fig. S3).

Automated image analysis for quantification of fluorescence expression was performed using Visiopharm integrator system software version 2021.9 (VIS, Visiopharm, Denmark). Analysis was performed blinded. For quartile analyses, Q1 (0–25%), Q2 (26–50%), Q3 (51–75%) and Q4 (76–100%) were calculated based on the number of cells corresponding to a phenotype for each sample. For median analyses, low vs high groups were divided based on the median number of cells (0–50% vs 51–100%). Unless indicated otherwise (Figs. S4 and S9), the cut-off values calculated for all breast cancer types were also used to stratify TNBC samples into groups. If more than 25% (for quartiles) or 50% (for median) of the samples had the same value, the groups were modified to include all samples with the same value.

### Statistical analyses

For this study, we excluded cores with a total area inferior to 50,000µm^2^, as well as the ones that had less than 5% of surface epithelium (as determined by CK8-18-positive area) or less than 500 cells based on DAPI stain. Also, samples for which less than 2 out of 3 cores were left after processing and following our exclusion criteria were not considered in our statistical analysis. Means of replicates for each sample were calculated and used for analysis.

Groups were compared using the Mann–Whitney or ANOVA tests from GraphPad Prism software (V6, GraphPad, La Jolla, CA) as indicated in the figure legends. Statistical survival and disease progression analyses were performed using the SPSS Statistics software (v25.0, SPSS Inc., Chicago, IL). Survival, progression and incidence were analysed using the Kaplan–Meier method and the log-rank test was used to measure statistical significance. *p*-values < 0.05 were considered statistically significant.

### Western blot analysis

Murine lymphocytic leukemia L1210 and breast carcinoma 4T1 cells (both from ATCC) were cultured in DMEM medium (Gibco, Waltham, MA, USA) supplemented with 10% FBS (Cytiva, Marlborough, UK) and kept at 37 °C with 5% CO_2_. PSMB9 and PMSB8 were knocked out using the CRISPR-Cas9 system pSpCas9(BB)-2A-GFP vector (PX458, Addgene, Watertown, MA). Briefly, cells were transfected using lipofectamine 2000 (11668019, Thermofisher), GFP + cells were then sorted and clones were isolated and expanded. Wildtype and knockout cells were treated with IFNγ (315-05-100 μg, Peprotech, Cranbury, NJ) at a concentration of 200 pg/µL for 24 h prior to sample collection. For Western blot analysis, cell lysates were generated using RIPA buffer (25 mM Tris–HCl pH 7.6, 150 mM NaCl, 5 mM EDTA, 1% Triton X-100, 1% sodium deoxycholate, 0.1% SDS) supplemented with complete protease inhibitors (11836153001, Sigma-Aldrich). Samples were migrated on 12% gels and transferred onto 0.45 µm nitrocellulose membranes (1620115, Biorad). Membranes were blocked in milk (5%) and probed with rabbit anti-mouse PSMB8 (13635 diluted 1:500, Cell Signaling Technology, Danvers, MA), rabbit anti-mouse PSMB9 (ab3328 diluted 1:250, Abcam) or rabbit anti-mouse GAPDH (2118 diluted 1:1000, Cell Signaling Technology), as well as goat anti-rabbit-HRP IgG (7074S diluted 1:2000, Cell Signaling Technology). Signals were revealed using the immobilon forte Western HRP substrate (WBLUF0500, Millipore Sigma) and with a Chemidoc imaging system (Biorad).

## Supplementary Information

Supplementary Information 1.

Supplementary Information 2.

## Data Availability

The data used and/or analysed during the current study available from the corresponding author on reasonable request.
