# Supplementary material for: Increased expression of the immunoproteasome subunits PSMB8 and PSMB9 by cancer cells correlate with better outcomes for triple-negative breast cancers
Source: Sci Rep. 2023 Feb 6;13:2129. doi: 10.1038/s41598-023-28940-2 (PMC9902398; doi:10.1038/s41598-023-28940-2)
Supplement: Supplementary file 1 — Supplementary Information 1. [file 41598_2023_28940_MOESM1_ESM.pdf]

TABLE 1

	Characteristics	Total population	TNBC
**Number of patients**		2070	282
**Surgery**	Median age (years)	58 (24 – 91)	55 (27 – 91)
**Follow-up**	Median (months)	117 (2 – 253)	131 (2 – 245)
**Morphology**	Ductal	1613	227
	Lobular	223	6
	Mucinous	50	1
	Medullary	28	19
	Papillary	11	2
	Tubular	16	0
	Others	129	27
**Grade**	1	352	6
	2	939	46
	3	776	229
**pTNM**	I	1060	106
	II	899	151
	III	88	20
	IIII	21	5
**Positive margin**		80	5
**Family history**	Breast cancer	572	77
	Ovarian cancer	56	4
	Other cancer	474	50
**Adjuvant therapies**	Chemotherapy	1169	223
	Hormonotherapy	1483	10
	Targeted therapy	247	4
	Radiotherapy	1596	223
	Other	23	6
**Other**	ER expression	1552	
	PR expression	1384	
	HER2 expression	291	
	Menopause	1342	170
	Gravida (>0)	1441	203
	Parity (>0)	1378	196
	Aborta (>0)	464	59

TABLE 2

Figure	Markers	Group	Range of positive-cells	All n	TNBC n
Fig. 2, S5	PSMB8 - median	low	0 - 212.33	935	101
		high	212.34 - 3591	934	165
	PSMB9 - median	low	0 - 63.66	881	94
		high	63.67 - 2517.66	879	169
	PSMB8 - quartiles	0-25%	0 - 56.66	465	43
		25-50%	56.67 - 212.33	467	57
		50-75%	212.34 - 653.33	467	57
		75-100%	653.34 - 3591	467	108
	PSMB9 - quartiles	0-25%	0 - 15.33	442	53
		25-50%	15.34 - 63.66	442	41
		50-75%	63.67 - 203.33	439	57
		75-100%	203.34 - 2517.66	440	112
	CD45 - median	low	0 - 0.5	1049	205
		high	0.51 - 128.33	861	64
Fig. 7, S10	PSMB8+ CK8-18+ - median	low	0 - 70.33	933	79
		high	70.43 - 2277.33	934	187
	PSMB8+ CK8-18- - median	low	0 - 78.33	934	144
		high	78.34 - 2830.33	933	122
	PSMB8+ CD45+ - median	low	0	1639	236
		high	0.1 - 8.33	230	30
	PSMB9+ CK8-18+ - median	low	0 - 7.66	884	60
		high	7.67 - 1068.33	879	203
	PSMB9+ CK8-18- - median	low	0 - 45.33	881	129
		high	45.34 - 1637.33	878	133
	PSMB9+ CD45+ - median	low	0	1634	248
		high	0.1 - 21	129	15
Fig. 8	PSMB8+ CK8-18+ - median	low	0 - 70.33	-	53
		high	70.43 - 2277.33	-	167
	PSMB9+ CK8-18+ - median	low	0 - 7.66	-	42
		high	7.67 - 1068.33	-	176
Fig. S4	PSMB8 - median	low	0 - 431.32	-	133
		high	431.33 - 3445.67	-	133
	PSMB9 - median	low	0 - 117.83	-	132
		high	117.84 - 2517.67	-	131
	PSMB8 - quartiles	0-25%	0 - 94.33	-	66
		25-50%	94.34 - 431.32	-	67
		50-75%	431.33 - 1077.66	-	66
		75-100%	1077.67 - 3445.67	-	67
	PSMB9 - quartiles	0-25%	0 - 28.25	-	66
		25-50%	28.26 - 117.83	-	66
		50-75%	117.84 - 498.25	-	65
		75-100%	498.26 - 2517.67	-	66
	PSMB8 - Q1 vs Q2-3-4	0-25%	0 - 94.33	-	66
		25-100%	94.34 - 3445.67	-	200
	PSMB9 - Q1 vs Q2-3-4	0-25%	0 - 28.25	-	66
		25-100%	28.26 - 2517.67	-	197
Fig. S9	PSMB8+CK8-18+ - median	low	0 - 217.5	-	133
		high	217.51 - 1890.33	-	133
	PSMB9+CK8-18+ - median	low	0 - 51.83	-	132
		high	51.84 - 1068.33	-	131
	PSMB8+CK8-18+ - quartiles	0-25%	0 - 48.34	-	67
		25-50%	48.35 - 217.5	-	66
		50-75%	217.51 - 653	-	67
		75-100%	653.1 - 1890.33	-	66
	PSMB9+CK8-18+ - quartiles	0-25%	0 - 9	-	66
		25-50%	9.1 - 51.83	-	66
		50-75%	51.84 - 195.125	-	65
		75-100%	195.126 - 1068.33	-	66
	PSMB8+CK8-18+ - Q1 vs Q2-3-4	0-25%	0 - 48.34	-	67
		25-100%	48.35 - 1890.33	-	199
	PSMB9+CK8-18+ - Q1 vs Q2-3-4	0-25%	0 - 9	-	66
		25-100%	9.1 - 1068.33	-	197

FIGURE S1

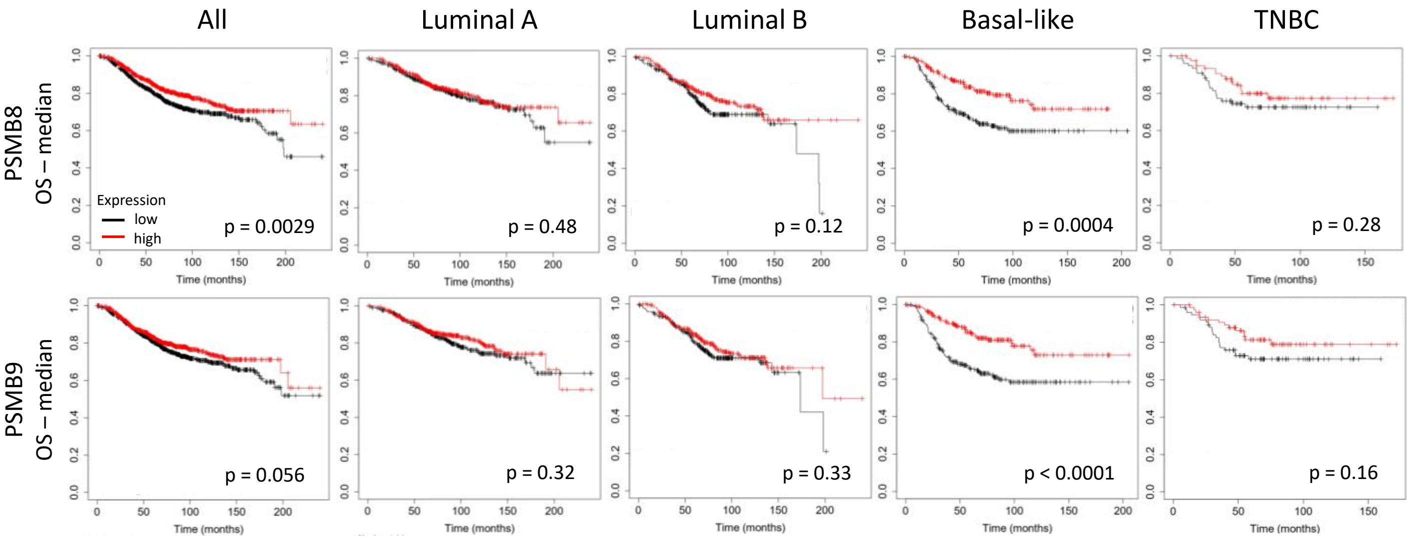
All
Luminal A
Luminal B
Basal-like
TNBC
PSMB8
OS – median
PSMB9
OS – median
Expression
low
high
p = 0.0029
p = 0.48
p = 0.12
p = 0.0004
p = 0.28
p = 0.056
p = 0.32
p = 0.33
p < 0.0001
p = 0.16
Time (months)

FIGURE S2

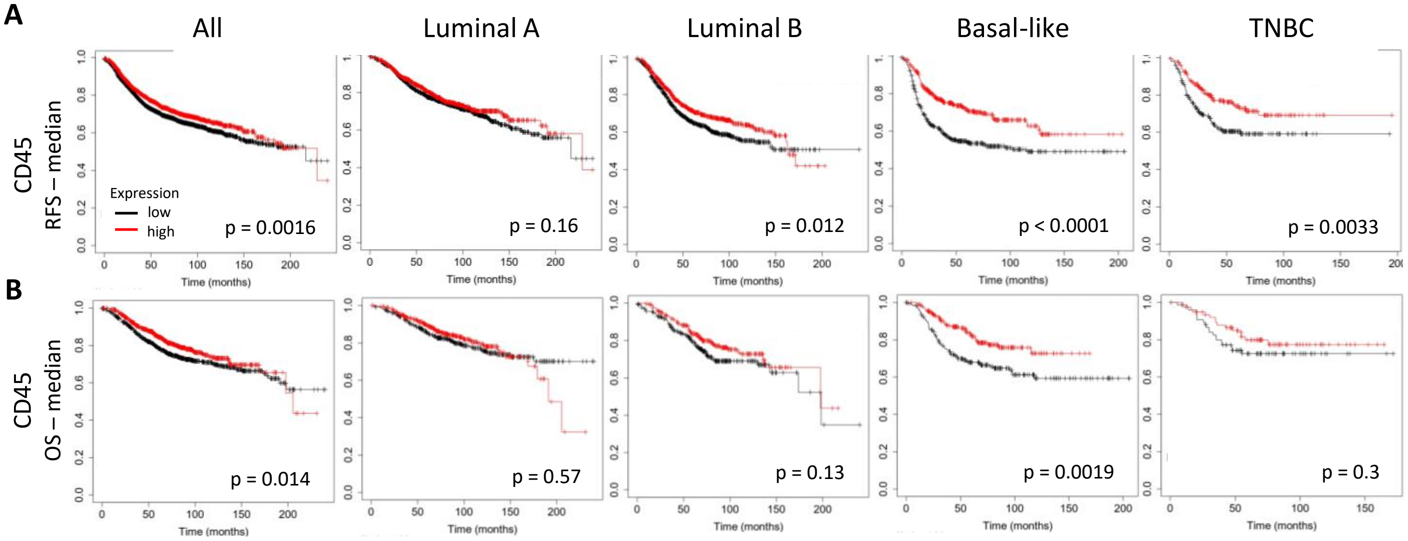

A
All
Luminal A
Luminal B
Basal-like
TNBC
CD45
RFS – median
Expression
low
high
p = 0.0016
p = 0.16
p = 0.012
p < 0.0001
p = 0.0033
Time (months)
B
CD45
OS – median
p = 0.014
p = 0.57
p = 0.13
p = 0.0019
p = 0.3

FIGURE S3

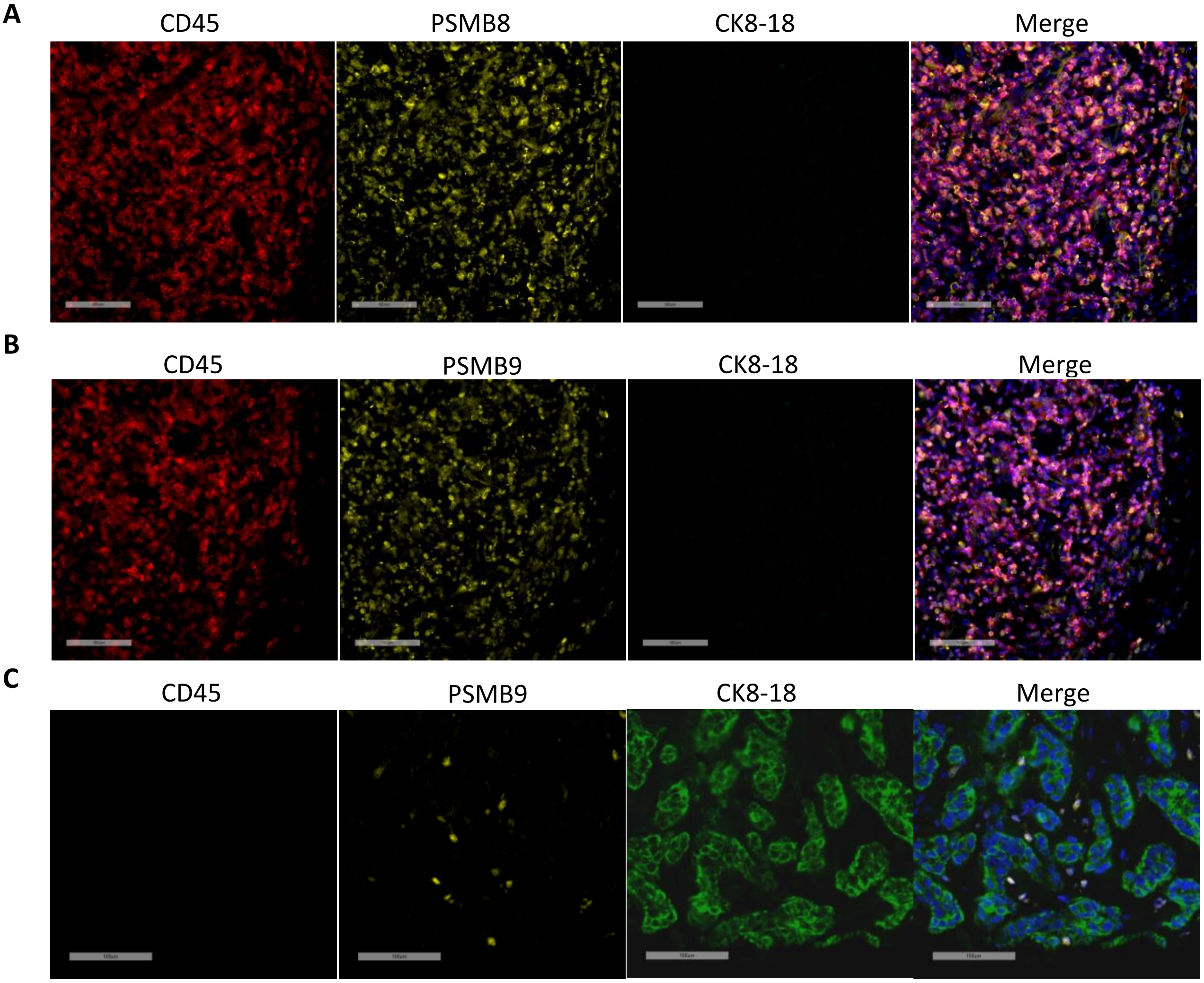
A
CD45
PSMB8
CK8-18
Merge
B
CD45
PSMB9
CK8-18
Merge
C
CD45
PSMB9
CK8-18
Merge

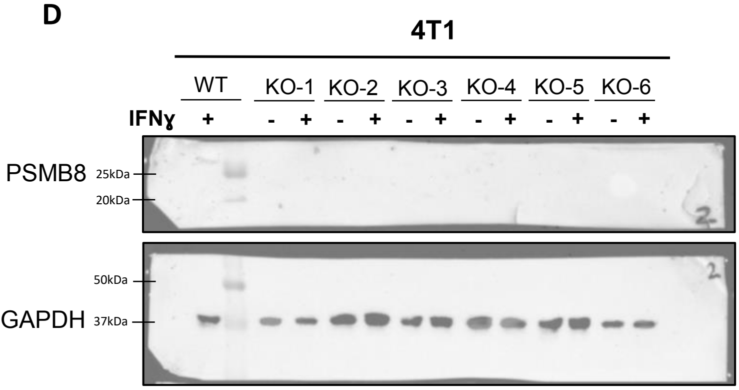
D
4T1
WT
KO-1
KO-2
KO-3
KO-4
KO-5
KO-6
IFNγ
+ - + - + - + - + - + - +
PSMB8
25kDa
20kDa
50kDa
GAPDH
37kDa

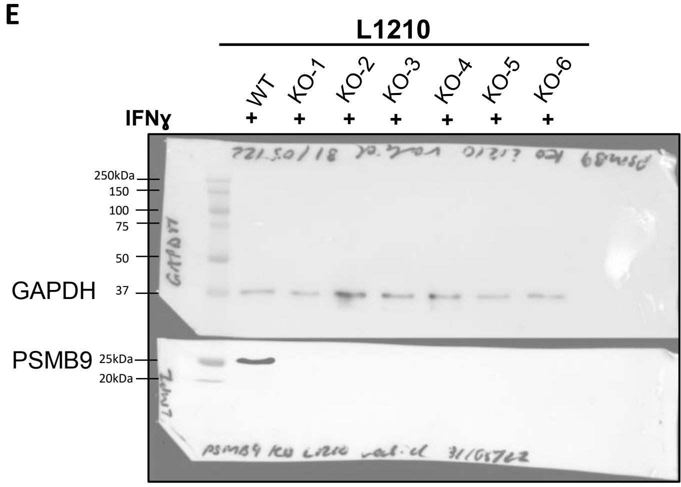
E
L1210
WT
KO-1
KO-2
KO-3
KO-4
KO-5
KO-6
IFNγ
+ + + + + + +
250kDa
150
100
75
50
GAPDH
37
PSMB9
25kDa
20kDa

FIGURE S4

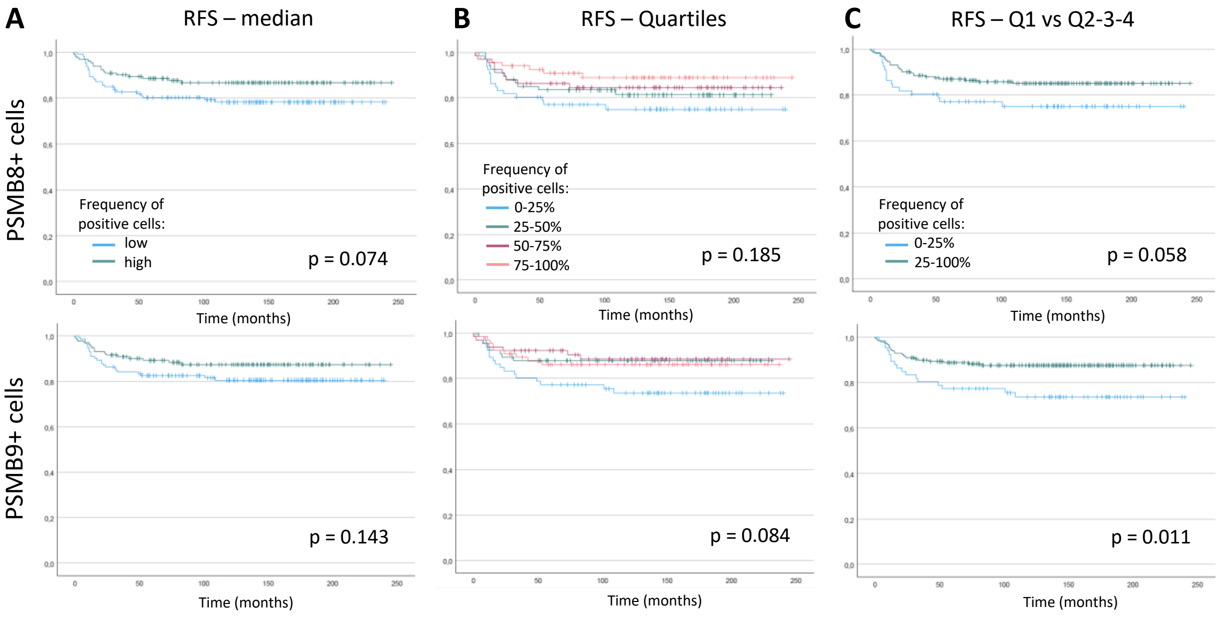

A
RFS – median
B
RFS – Quartiles
C
RFS – Q1 vs Q2-3-4
PSMB8+ cells
PSMB9+ cells
Frequency of positive cells:
low
high
0-25%
25-50%
50-75%
75-100%
25-100%
p = 0.074
p = 0.185
p = 0.058
p = 0.143
p = 0.084
p = 0.011
Time (months)

FIGURE S5

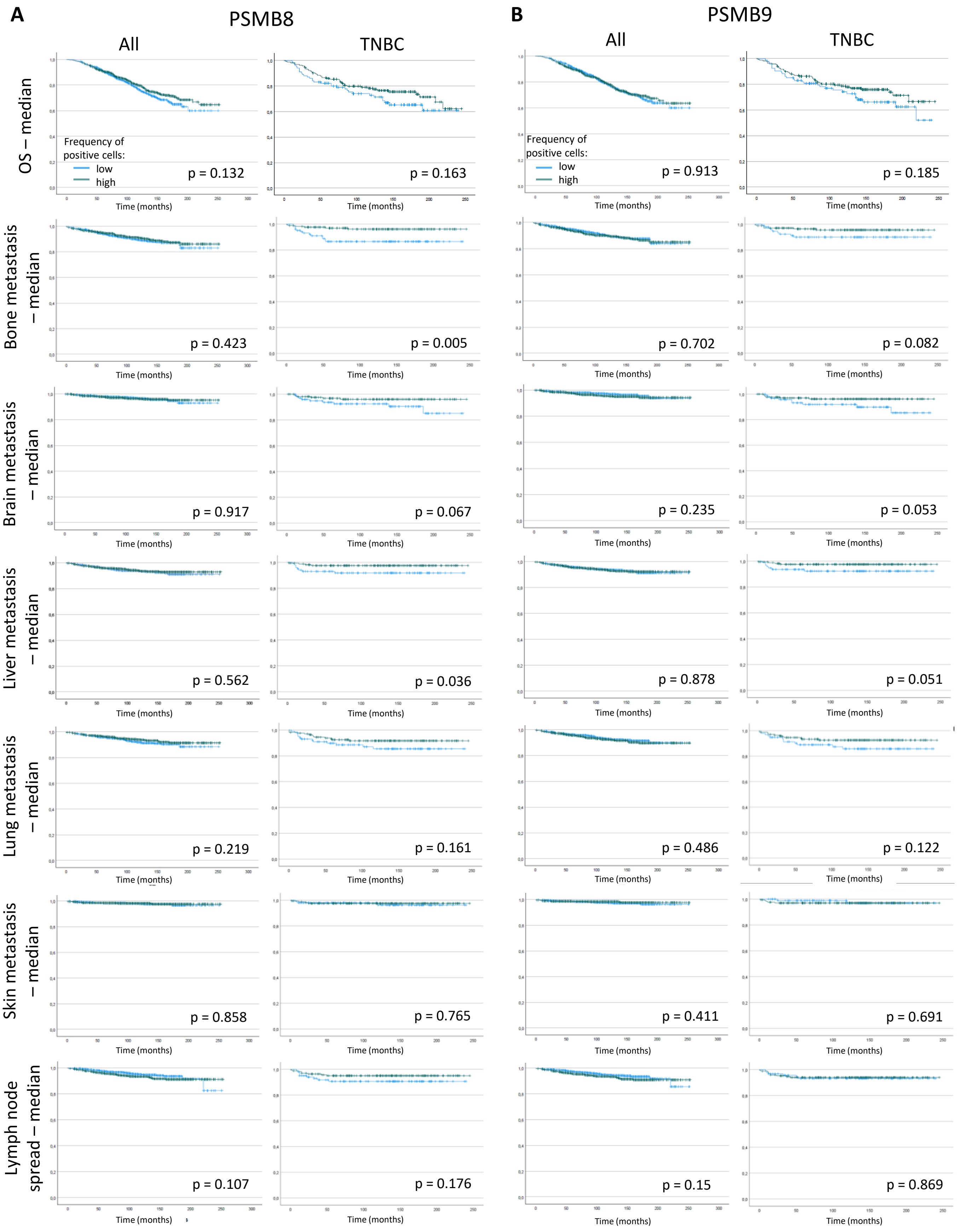
A
PSMB8
All
TNBC
B
PSMB9
All
TNBC
OS – median
Bone metastasis – median
Brain metastasis – median
Liver metastasis – median
Lung metastasis – median
Skin metastasis – median
Lymph node spread – median
Frequency of positive cells:
low
high
Time (months)
p = 0.132
p = 0.163
p = 0.913
p = 0.185
p = 0.423
p = 0.005
p = 0.702
p = 0.082
p = 0.917
p = 0.067
p = 0.235
p = 0.053
p = 0.562
p = 0.036
p = 0.878
p = 0.051
p = 0.219
p = 0.161
p = 0.486
p = 0.122
p = 0.858
p = 0.765
p = 0.411
p = 0.691
p = 0.107
p = 0.176
p = 0.15
p = 0.869

FIGURE S6

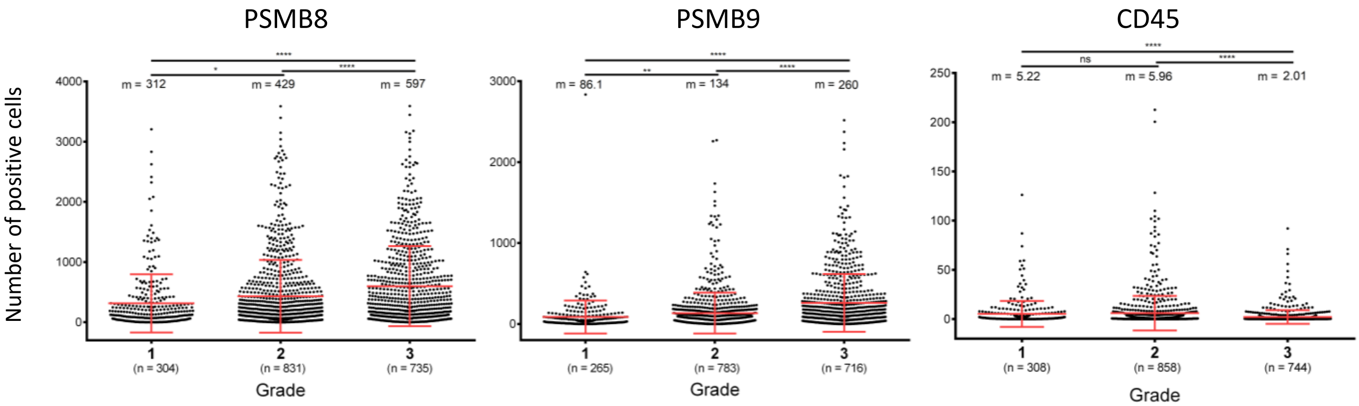
PSMB8
PSMB9
CD45
Number of positive cells
m = 312
m = 429
m = 597
m = 86.1
m = 134
m = 260
m = 5.22
m = 5.96
m = 2.01
ns
1 (n = 304)
2 (n = 831)
3 (n = 735)
1 (n = 265)
2 (n = 783)
3 (n = 716)
1 (n = 308)
2 (n = 858)
3 (n = 744)
Grade
Grade
Grade

FIGURE S7

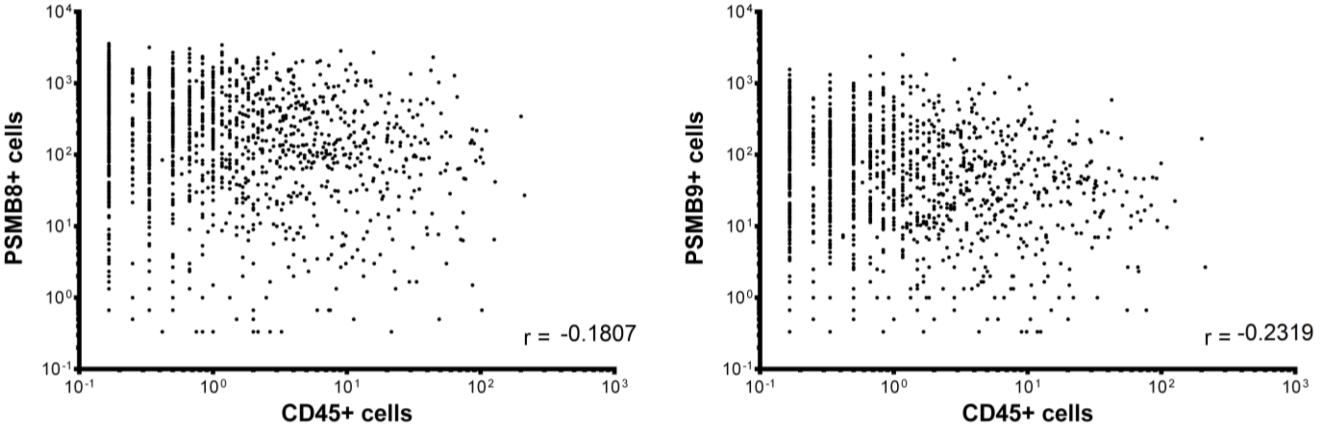

PSMB8+ cells
CD45+ cells
r = -0.1807
PSMB9+ cells
CD45+ cells
r = -0.2319

FIGURE S8

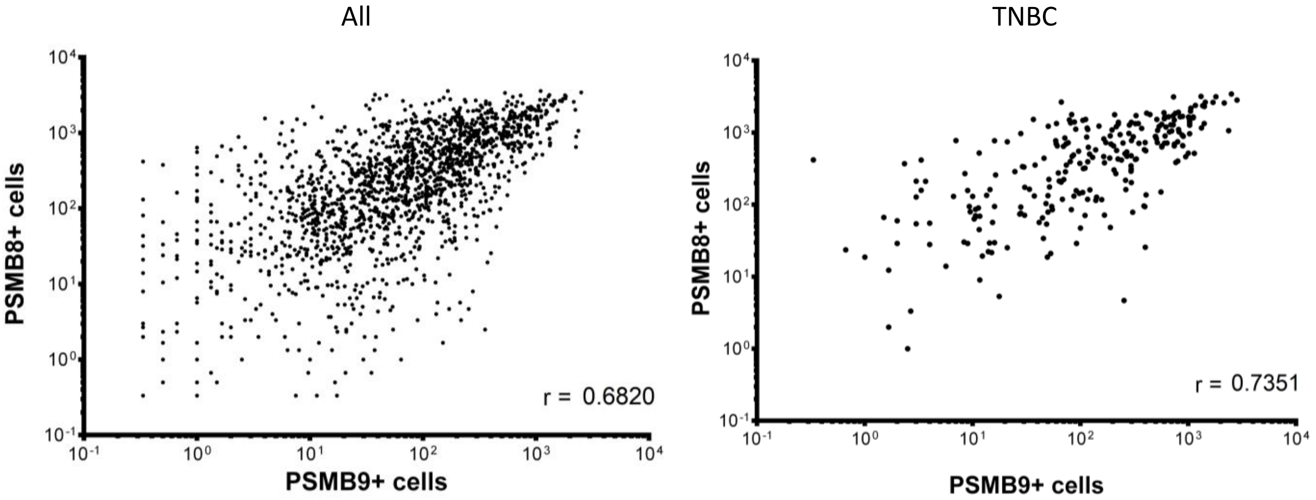

FIGURE S9

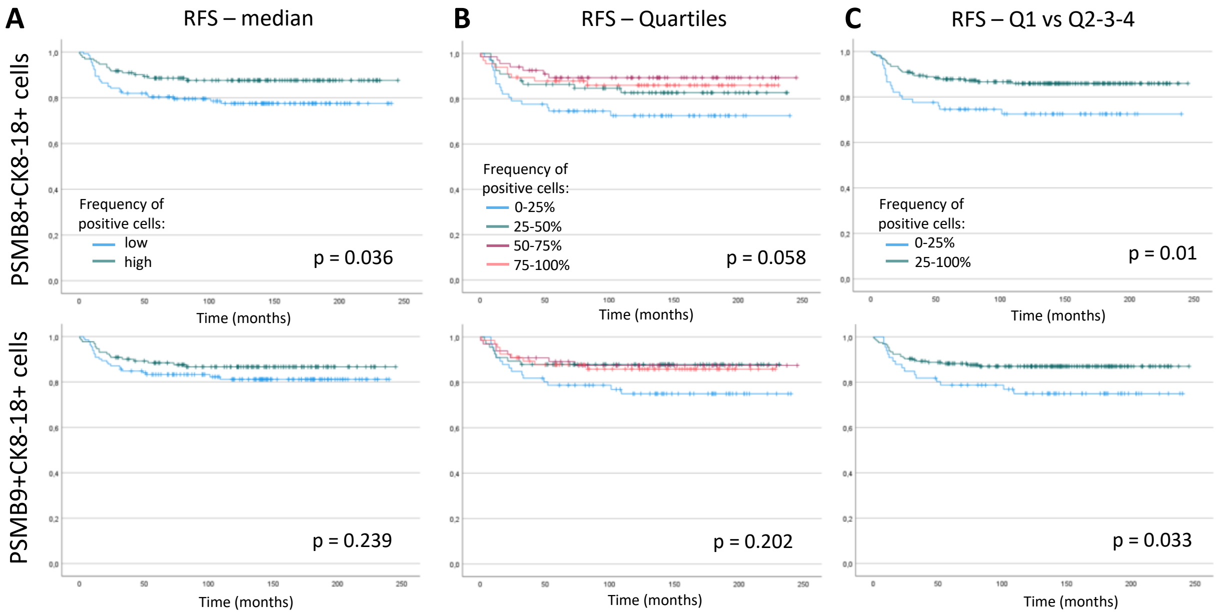

A
RFS – median
B
RFS – Quartiles
C
RFS – Q1 vs Q2-3-4
PSMB8+CK8-18+ cells
PSMB9+CK8-18+ cells
Frequency of positive cells:
low
high
0-25%
25-50%
50-75%
75-100%
0-25%
25-100%
p = 0.036
p = 0.058
p = 0.01
p = 0.239
p = 0.202
p = 0.033
Time (months)

FIGURE S10

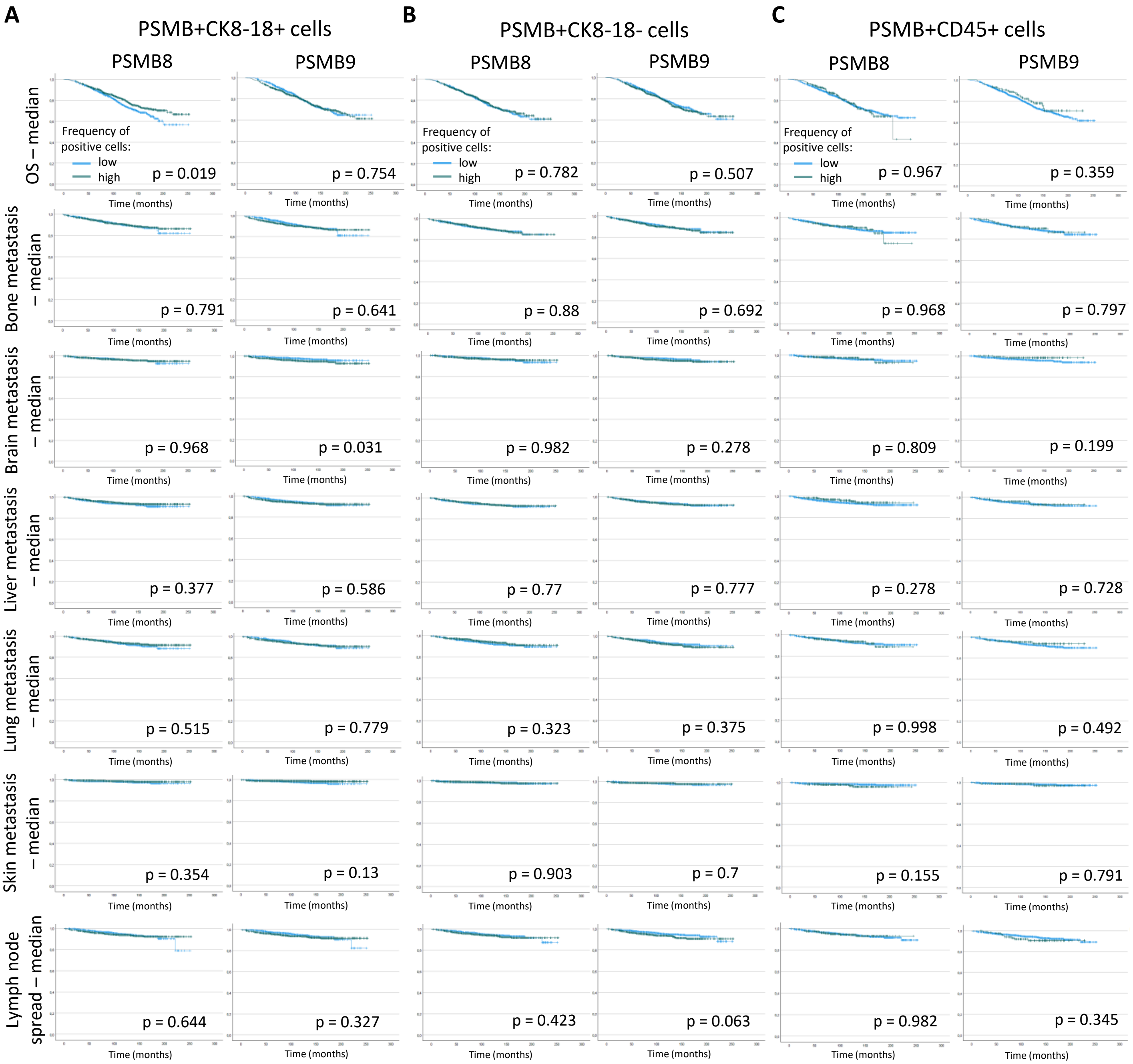
A
PSMB+CK8-18+ cells
B
PSMB+CK8-18- cells
C
PSMB+CD45+ cells
PSMB8
PSMB9
PSMB8
PSMB9
PSMB8
PSMB9
OS – median
Frequency of positive cells:
low
high
p = 0.019
p = 0.754
p = 0.782
p = 0.507
p = 0.967
p = 0.359
Bone metastasis – median
p = 0.791
p = 0.641
p = 0.88
p = 0.692
p = 0.968
p = 0.797
Brain metastasis – median
p = 0.968
p = 0.031
p = 0.982
p = 0.278
p = 0.809
p = 0.199
Liver metastasis – median
p = 0.377
p = 0.586
p = 0.77
p = 0.777
p = 0.278
p = 0.728
Lung metastasis – median
p = 0.515
p = 0.779
p = 0.323
p = 0.375
p = 0.998
p = 0.492
Skin metastasis – median
p = 0.354
p = 0.13
p = 0.903
p = 0.7
p = 0.155
p = 0.791
Lymph node spread – median
p = 0.644
p = 0.327
p = 0.423
p = 0.063
p = 0.982
p = 0.345
Time (months)
