# Supplementary material for: Increased expression of the immunoproteasome subunits PSMB8 and PSMB9 by cancer cells correlate with better outcomes for triple-negative breast cancers
Source: Sci Rep. 2023 Feb 6;13:2129. doi: 10.1038/s41598-023-28940-2 (PMC9902398; doi:10.1038/s41598-023-28940-2)
Supplement: Supplementary file 2 — Supplementary Information 2. [file 41598_2023_28940_MOESM2_ESM.docx]

**Supplementary Table 1. Clinical information of the Quebec breast cancer foundation (QBCF) cohort.**

The cohort includes 2070 patient samples, 282 of which are TNBCs. Patients were followed up to 131 months after their diagnosis and presented different morphologies, grades (ranging from 1 to 3) and stages (pTNM classification) of the disease. All patients underwent surgery and most of them received adjuvant therapies. Additional information is also detailed.

**Supplementary Table 2. Ranges of number of marker-positive cells for each figure according to subpopulation analyzed.**

Data was segregated according to median, quartiles of expression or the presence or absence of cell populations in the different figures. The details for each panel of all figures analyzed at the protein level can be found here.

**Figure S1. High PSMB8 and PSMB9 mRNA expression correlates with better survival in basal breast cancer.**

OS according to PSMB8 and PSMB9 mRNA expression of all breast cancer patients together, as well as for the luminal A, luminal B, basal and TNBC subtypes individually. The groups were divided according to their median expression. The red and black lines correspond to high and low expression, respectively. Statistical analyses by Logrank test: differences are considered statistically significant when p < 0.05.

**Figure S2. High CD45 mRNA expression is associated with better outcomes in basal breast cancer and TNBC.**

Kaplan-Meier analyses of the (A) RFS and (B) OS of all breast cancers, as well as for the luminal A, luminal B, basal or TNBC subtypes. The groups were divided according to their median CD45 mRNA expression. The red and black lines correspond to high and low expression, respectively. Statistical analyses by Logrank test: differences are considered statistically significant when p < 0.05.

**Figure S3. Multi-color IF panels used in this study.**

Paraffin-embedded human lymph node samples were stained with combinations of CD45 (red), CK8-18 (green), DAPI (blue) and (A) PSMB8 (yellow) or (B) PSMB9 (yellow). (C) Paraffin-embedded human breast cancer samples stained with the same antibody panel as in (B). Scale bar = 100µm. (D) Western blot analysis of 4T1 wildtype versus 4T1 PSMB8 K-O cells and (E) L1210 wildtype versus PSMB9 K-O cells treated with IFNγ to induce ImP expression. GAPDH was used as a loading control.

**Figure S4. High PSMB8 and PSMB9 protein expression is associated with better RFS in TNBC.**

RFS according to the amount of PSMB8- and PSMB9-expressing cells of TNBC patients (upper and lower panel, respectively). Samples were divided according to (A) their median, (B) quartiles of expression or (C) when comparing the lower quartile to others. Quartiles and medians we calculated based on the range of positive cells found in the TNBC cohort. For median expression, as well as Q1 vs others, the green and blue lines correspond to high and low frequency of positive cells, respectively; for quartiles analysis, blue, green, purple and red lines correspond to the lowest to highest frequency of positive cells, respectively. Statistical analyses by Logrank test: differences are considered statistically significant when p < 0.05.

**Figure S5. High PSMB8 and PSMB9 protein expression is associated with a trend to better OS and less metastasis in TNBC specifically.**

Kaplan Meier analyses of, from top to bottom, the OS, as well as the development of bone, brain, liver, lung, skin metastases and spread to lymph nodes of all breast cancer subtypes and TNBC patients according to the median number of (A) PSMB8- or (B) PSMB9-expressing cells. The green and blue lines correspond to high and low frequency of positive cells, respectively. Statistical analyses by Logrank test: differences are considered statistically significant when p < 0.05.

**Figure S6. Overall quantity of PSMB8- and PSMB9- expressing cells correlate with breast cancer grades.**

Quantification of the amount of cells positive for PSMB8, PSMB9 or CD45 and segregated according to the grade of the disease. The number of samples from each grade included in the analysis is indicated in brackets below the corresponding group. Statistical analyses by Mann-Whitney test, ns: p > 0.05, *: p ≤ 0.05, **: p ≤ 0.01, ****: p ≤ 0.0001.

**Figure S7. PSMB8- or PSMB9- and CD45-expressing cells do not correlate.**

Linear regression correlation analysis of the quantification of PSMB8- or PSMB9- and CD45-positive cells. Spearman's rank correlation coefficient indicates a correlation when r ≥ 0.2.

**Figure S8. Overall quantities of PSMB8 and PSMB9 cells correlate together.**

Linear regression correlation analysis of the number of PSMB8- and PSMB9-positive cells in all breast cancer subtypes together and TNBC samples. Statistical analyses by nonparametric Spearman's rank correlation coefficient indicates a correlation when r ≥ 0.2.

**Figure S9. High numbers of PSMB8- and PSMB9- expressing tumor cells is associated with better prognosis in TNBC samples.**

RFS according to the amount of PSMB8- and PSMB9-expressing CK+ cells of TNBC patients (upper and lower panels, respectively). Samples were divided according to (A) their median, (B) quartiles of expression or (C) when comparing the lower quartile to others. Quartiles and medians we calculated based on the range of positive cells found in the TNBC cohort. For median expression, as well as Q1 vs others, the green and blue lines correspond to high and low frequency of positive cells, respectively; for quartiles analysis, blue, green, purple and red lines correspond to the lowest to highest frequency of positive cells, respectively. Statistical analyses by Logrank test: differences are considered statistically significant when p < 0.05.

**Figure S10. Protein expression of PSMB8 and PSMB9 by tumor, stromal and immune cells do not correlate with outcomes in all breast cancers.**

Kaplan Meier analyses of, from top to bottom, the OS, as well as the onset of bone, brain, liver, lung, skin metastases and spread to lymph nodes of all breast cancer patients according to the median quantity of PSMB8 or PSMB9 at the protein level in (A) tumor, (B) stromal or (C) immune cells. The green and blue lines correspond to high and low frequency of positive cells, respectively. Statistical analyses by Logrank test: differences are considered statistically significant when p < 0.05.
